# Twenty-Third Annual Session of the Georgia State Dental Society

**Published:** 1891-10

**Authors:** 


ARTICLE II.

### TWENTY-THIRD ANNUAL SESSION OF THE GEORGIA STATE DENTAL SOCIETY.

WAS HELD IN THE ASSEMBLY ROOM AT ST. SIMON'S HOTEL, ST. SIMON'S ISLAND, GEORGIA, ON THE 17TH OF MAY, 1891.

[CONTINUED FROM PAGE 49.]

EVENING SESSION.

Meeting was called to order at 8:30 p. m,, May 29th, 1891, Dr. Adair, President, in the chair.

Dr. Colding.—With reference to the disposition of the minutes, I have seen Mr. Selby and Dr. Johnson. Mr. Selby says he has not the authority to purchase them, and Dr. Johnson says that if the Convention will give them to the Southern Dental Journal for publication he will mail each member a copy containing the proceedings of the Convention; and the Southern Dental Journal makes that proposition to this Convention.

Upon motion, the proposition was accepted, and the Secretary instructed to deliver the minutes to Dr. Johnson for publication.

Dr. Atkinson then offered the following resolution:

WHEREAS, The Southern Dental Journal has for ten years advocated the highest and best interests of this society, and has at all times proclaimed the honor and upheld the dignity of the dental profession.

*Be it resolved*, That we do hereby again confirm and constitute the said journal as the *offical organ* of this society, and it is hereby ordered that the proceedings of the Convention be turned over to said journal for publication.

Resolution unanimously adopted.

Dr. Colding.—The Executive Committee would like at this point of the meeting to present the names of Dr. F. C. Wilson, of Savannah, and Dr. G. B. Moore, of Columbus, for membership in the Georgia State Dental Society.

Upon motion, the rules were suspended and the applicants elected by acclamation.

Dr. Holland.—I move that all members in arrears be reinstated upon their paying all arrearage due by them, and dues for the present year.

Motion seconded and carried.

Dr. Carpenter.—Relative to this matter of county tax, when a dentist goes into another practice, or is called there, he has to pay a tax of ten dollars, or he is considered an itinerant. I was in the office of the Comptroller-General the other day, and was talking about this matter, and he said to Dr. Thornton and myself that the best course for our society to pursue in this matter is to have a case carried to the Supreme Court and decided. And Dr. Thornton was in my office a day or two later, and said to me that if the question was brought up before this Convention, to say to the Convention that if the society would help him a little he would be made a subject in a case to go to the Supreme Court. All Dr. Thornton desires is that this society assist him in paying the costs of having this matter pushed through to a final decision by the Supreme Court; and I simply lay the matter before this society as a matter of grave interest to us all.

Dr. White.—I move that this society assist Dr. Thornton in this case to the amount of fifty dollars.

Motion was lost, and a committee appointed to present the matter to the Legislature.

Dr. Carpenter has paid one tax of ten dollars and is threatened with another. This is a special tax that every dentist in the State of Georgia has to pay whether he goes over in another county other than the one in which he has taken out a license or not.

Upon motion the following committee was appointed, with President Adair chairman and ex-officio member of the committee, to look into the matter fully and see what could be done to relieve the dentists of Georgia from this unjust tax, and see what could be done in repealing the law.

L. D. Carpenter, B. H. Catching, Frank Holland, Committee.

Dr. Colding.—I move that each member of this society call on his representative and invoke his co-operation in the furtherance of this matter.

Seconded and carried.

Upon motion,-meeting then adjourned.

---

### AFTERNOON SESSION.

Meeting was called to order at 2:30 p. m., May 21st, 1891, with President Adair in the chair.

### PULPLESS TEETH.

Dr. Atkinson —In regard to the heroic treatment of pulpless teeth by boring through the gum to the root of the tooth, in nearly all cases, and pumping through that into the tooth—I should like to know what degree of success there is in that treatment and how often it is done and under what conditions it would be resorted to.

Dr. Colding.—I have treated teeth in this manner occasionally, but not going back further than the bi-cuspid teeth.

Dr. Atkinson.—Dr. Carpenter, you are familiar with such treatment and I would like to get some information on that point from you; if you do this, under what conditions, and what extent of success do you have, if you find a tooth in the mouth is dead? The arguments brought me are that it would be advisable to drill a hole through and put medicines in it, stopping fistula.

Dr. Carpenter.—I do not think I ever drilled through one in my life. I have been in the habit of removing decay, when the trouble exists in the six front upper teeth, from nerve cavity of tooth, cleansing it as thoroughly as I can, then forcing creosote or carbolic acid into same (creosote being my preferance), taking a little probe, winding cotton around it, dipping it in creosote and pumping it through canal until I can see it come out, then stop apex of root of tooth with gold or any other material which I might think desirable to use, and in almost every instance nature will go along and take care of itself, and you can fill the tooth at some future sitting; this is where there is a fistulous opening. Where there is a blind abscess, I should be very careful in removing the decay and very gentle in passing up the nerve canal, and would leave it for a day or two before filling same, so the irritation from the tooth could entirely subside. For quite a number of years my experience has been, with bicuspids and molars, that where a bad decay is and nerve is dead, you may find teeth very offensive and in a very bad condition, but I have repeatedly removed that decayed substance and that superfluous growth of tissue, and cleansing canal as good as I could, and then filling the tooth, and sometimes it would be so sensitive that the patient would jump with pain.

I will state that there is a great majority of cases coming under my supervision of this character pass away in a day or two, and being filled then and there, in most instances, have proved a success. It is true that you may expect occasionally to have one go back on you, but my observation is it will be very seldom.

The President.—Dr. Browne in the Chair. I had a young lady patient where the four incisor teeth were all fistulous, and I pumped cresote in them and kept pumping it in them, and I filled those teeth that very day. (Giving illustration on blackboard.)

Dr. Atkinson.—With what material did you stop that root, Dr. Adair?

Dr. Adair.—With gold.

Dr. Williams.—Dr. Carpenter, what is the treatment for abscesses after you have put on crown?

Dr. Carpenter.—I have no better treatment than tincture of aconite and iodine.

Dr. Colding.—I have had some little trouble in the same direction, and used compound tincture absinthe.

Dr. Fogg.—I will endorse what Dr. Carpenter has said on this subject. I have had a little experience with fistulous openings through the nerve canal, and those cases have given no little trouble, but I have succeeded in stopping them by lancing the gum.

Dr. Carpenter.—If you will stop the foramen at apex of the tooth, you give nature a chance to work, but if you go in there and tear out everything nature has done you cannot expect the tooth to get along as well as when nature takes care of itself.

Dr. Adair.—I make the assertion that you cannot cure a chronic abscess where it has been running, with one application of carbolic acid; when you have necrosis, carbolic acid will not cure it up alone and with one application.

Dr. Carpenter.—I make the assertion that there is no necrosis under ordinary conditions; it might be caries but not necrosis.

Dr. Browne.—It seems to me that upon this question of treatment of abscesses of teeth, we should come to some conclusion as to fistulous opening, but when you get one without a fistulous opening, what we term a blind abscess, then there is greater difficulty. I have been studying that subject for some years, and reading all that I could get on it, and am beginning to think that we ought to get down to some definite treatment.

After I have got the root thoroughly disinfected and dried I will fill it with oxy-chloride of zinc, on a shred of cotton.

Dr. Carpenter.—If you pack the nerve canal firm as you can with cotton clear to the apex of the tooth until you can prepare root of tooth, what would be the objection to letting cotton stay there, and fill the tooth?

Dr. Browne.—If that cotton was perfectly packed I would have no objection to leaving it there, and where I find anything like a large opening through the foramen, I always put a small piece of cotton in it and let it remain there to stop the foramen. The chloride is a purifier, so to speak.

Upon motion, subject was passed.

The Secretary then read a letter from Dr. B. H. Catching, of Atlanta, Ga., stating that he would be unable to attend the Convention, and stating further, that the Supreme Court of the United States had decided the Richmond patents held by the International Tooth Crown Company invalid.

The following resolution was offered by Dr. Browne:

WHEREAS, The Georgia State Dental Society, in session at St. Simon's Island, has heard with pleasure the decision of the United States Supreme Court in the Gaylord appealed case bearing upon the validity of the Richmond Crown; said court having decided the patent invalid.

"*Resolved*, That we extend to Dr. Crouse and co-laborers a rising vote of thanks for persistent efforts in the matter.

*Resolved, second*, That we heartily endorse the Dental Protective Association, and urge every member of the Georgia State Dental Society to become a member of it.

*Resolved, third*, That we urge the dentists of the United States not to relax but to increase their efforts to wipe out all such claims, which now materially affect our welfare."

Which resolution was unanimously adopted by a rising vote.

A letter was read from Prof. W. H. Morgan, stating he was very sorry he could not be at the Convention, giving as the cause of his non-attendance, ill health.

A committee consisting of Drs. Browne, Roach and Holmes, was appointed to draft and forward to J. N. Crouse, a resolution thanking him for his efforts in behalf of the Protective Association and the profession at large.

President.—What are we going to do, gentlemen, about printing ten thousand copies of Dr. Catching's address read last year at Gainesville?

Dr. White.—I move that the printing of this address be referred to the Executive Committee with power to act.

Motion was seconded and carried.

The Executive Committee reported the following names for membership:

Dr. T. P Hinman, Atlanta, Ga.

Dr. M. M. Pucket, Cartersville, Ga.

Dr. Hollinshead, Waycross, Ga.

Upon motion rules were suspended and applicants elected by acclamation.

Executive Committee made the following report in the case of Dr. Betts:

The Executive Committee recommends that our attorney in the case against Dr. Betts be instructed when the case is called for trial to respond as follows: That inasmuch as Dr. W. P. Betts has appeared before the Examining Board of the Georgia State Dental Society, and after passing a satisfactory examination has been granted a license by said board to practice dentistry in the State of Georgia, thus complying with the law, the Georgia State Dental Society requests that the case be dismissed; and see that it is entered upon the records of the court why the society asks for its dismissal.

Upon motion, the report of Executive Committee in the case of Dr. Betts was adopted.

Dr. Browne here exhibited Dr. Coyle's Reese mallet for the engine.

Committee appointed to prepare resolutions on the death of Dr. Atkinson, of New York, reported as follows:

WHEREAS, The members of the Georgia State Dental Society having heard with sincere regret of the death of W. H. Atkinson, M. D., D. D. S., of New York, an honorary member of this society, and one of the most eminent, learned, self-sacrificing and useful members of the dental profession; therefore be it

*Resolved*, That in the death of Dr. Atkinson the members of this society, in accord with the whole dental profession, feel not only a deep sense of personal sorrow, but they have been deprived of the teachings of one whose influence will be felt as long as time. While we bow in humble submission to the will of divine providence, keenly feeling our great loss at his sudden demise, it gives us pleasure to record our high appreciation of him. Be it further

*Resolved*, That these resolutions be entered on the minute book, published with the proceedings of the society, and that the secretary be requested to transmit a copy to the bereaved family of the deceased.

H. H. Johnson, W. R. Holmes, Richard Roach, Committee.

Upon motion, the above resolution was unanimously adopted by a rising vote.

Dr. Atkinson.—Your Committee on Clinics beg leave to report the following interesting clinics as having been made during the session.

Crown and Bridge Work, by H. H. Johnson, expressed in his own words as follows:

An extensive bridge including in its construction five teeth. The abutments to the bridge were the first or six year molar and lateral incisor, the canine remaining intact. A bar extending around it connecting the two parts of the bridge are given considerable support by having it served as an outward brace to break the force of an outward strain in biting. The suspended teeth were left central incisor and two left bicuspids. The first molar acting as abutment, was in a badly decayed and broken down condition, requiring to be built up with amalgam to preserve the form and then crowned with an all gold crown. The lateral forming the other abutment was in splendid condition and comparatively sound. The tooth was cut off and nerve driven out with a peg of orange wood. The operation was quickly performed and gave as little or less pain than one would ordinarly have expected. After cutting off the crown, the root was prepared in the usual way by paring off the walls until they were parallel to receive a band, the gold porcelain faced banded crown being used to form the anchorage at this point. The bar passing around the canine was make to cover as little of the inner surface of the tooth as possible, and passed above the gum margin in such a way as to be easily kept clean, the whole resulted in a strong and serviceable device.

Gold Filling—Knuckling, by Dr. Frank Holland, showing the very great importance of imitating nature.

Gold Filling, by Dr. Coyle, showing the Coyle Reese Mallet for the engine.

Amalgam Filling, by Dr. S. A. White, showing the value of self-cleansing surfaces.

Upon motion, the report of the Committee on Clinics was adopted.

---

ELECTION OF OFFICERS.

The following officers were elected to serve for the ensuing year:

Dr. W. G. Browne, President.

Dr. S. M. Roach, First Vice Prasident.

Dr. W. W. Hill, Second Vice-President.

Dr. L. D. Carpenter, Corresponding Secretary.

Dr. S. H. McKee, Recording Secretary.

Dr. H. A. Lawrence, Treasurer.

Board of Examiners consists of Dr. J. H. Coyle, Dr. A. G. Bouton, Dr. G. W. McElhaney, Dr. William C. Wardlaw and Dr. D. D. Atkinson.

The Executive Committee consists of Drs. H. S. Colding, H. H. Johnson, S. B. Adair, N. A. Williams and Frank Holland.

Place selected for next meeting is Rome, Ga.

Dr. McElhaney offered the following resolution:

*Resolved*, That the thanks of the "Georgia State Dental Society" are hereby tendered to Mr. J. H. Clark, the proprietor of the "Hotel St. Simons," for his uniform courtesy and attention to us during this meeting; and to Messrs. Selby and Brown for valuable and generous assistance to the clinics; to the Brunswick Daily Times for the many kind notices and published reports of this meeting; and to C. W. Deeming of the State Press; and to M. W. E. Kay, for their kind and attentive courties to this society; to Drs. Atkinson and Gale, of Brunswick, the local committee of arrangements, who having been so successful in making our meeting pleasant, and well remembered as one of the "good meetings" of the society.

The above motion was unanimously adopted.

After the installation of officers elected for the ensuing year, upon motion the meeting adjourned to meet in Rome, Ga., at such time as the executive committee may elect.—*Dental Luminary.*

---

ARTICLE III.

### BACTERIA OF THE HUMAN MOUTH.

